# Acceptability of Intimate Partner Violence among Male Offenders: The Role of Set-Shifting and Emotion Decoding Dysfunctions as Cognitive Risk Factors

**DOI:** 10.3390/ijerph16091537

**Published:** 2019-04-30

**Authors:** Ángel Romero-Martínez, Marisol Lila, Enrique Gracia, Christina M. Rodriguez, Luis Moya-Albiol

**Affiliations:** 1 Department of Psychobiology, University of Valencia, Valencia 46010, Spain; Luis.Moya@uv.es; 2 Department of Social Psychology, University of Valencia, Valencia 46010, Spain; Marisol.lila@uv.es (M.L.); Enrique.Gracia@uv.es (E.G.); 3 Department of Psychology, University of Alabama at Birmingham, Birmingham, AL 35233, USA; cmrpsych@uab.edu

**Keywords:** acceptability attitudes, cognitive deficits, emotion decoding, implicit measures, intimate partner violence, set-shifting

## Abstract

Attitudes towards the acceptability of intimate partner violence against women (IPVAW) contribute to an increased risk of IPVAW perpetration, and these attitudes are common among IPVAW offenders. Research suggests that IPVAW offenders present cognitive deficits related to information processing. Little is known, however, about how these deficits are related to the acceptability of IPVAW. The main aim of this study was to explore the relationship between specific cognitive deficits (i.e., deficits in attention switching, set-shifting, and emotion decoding abilities) and the acceptability of IPVAW in a sample of 84 IPVAW offenders. Results revealed that IPVAW offenders with deficits in attention switching, set-shifting, and emotion decoding abilities demonstrated greater acceptability of IPVAW, and these relationships remained significant after controlling for socio-demographic variables (i.e., age and educational level) and drug consumption. These results highlight the role of cognitive processes in maintaining attitudes of acceptability of IPVAW. Thus, the findings may guide professionals in developing specific intervention programs focused on improving cognitive abilities, in order to reduce the acceptability of IPVAW.

## 1. Introduction

Intimate partner violence against women (IPVAW) is a major public health problem and a violation of human rights of epidemic proportions. According to the World Health Organization [1], it is a major threat to the physical and psychosocial health of women victims, their children, and the broader community [2,3,4,5,6]. The lifetime prevalence of IPVAW in Western countries is estimated to be around 23% [7], ranging in the European Union between 13 and 32% [8,9]. According to various sources, the lifetime prevalence in Spain, where this study was conducted, is estimated to be around 13% [8,10,11]. 

The acceptability of violence in intimate relationships is one of the most relevant attitudes associated with the perpetration of IPVAW. Considerable research has shown that the acceptance and justification of IPVAW increase the risk of IPVAW perpetration, thus representing the main target for intervention programs [12,13,14,15,16,17,18,19]. As Martín-Fernández et al. noted, “high levels of acceptability of IPVAW can lead to the perception of this type of behavior as normative, increasing the risk of men perpetrating IPVAW” [20]. Perpetrators of intimate partner violence against women (IPVAW) tend to justify and accept this type of violence [20,21,22], and they tend to deny or minimize its consequences [23,24,25]. 

Additionally, research indicates that IPVAW offenders present cognitive deficits related to information processing. A growing body of empirical evidence suggests that IPVAW perpetrators, compared to non-violent individuals, are more likely to display several cognitive deficits in information processing, such as poor attention switching, set-shifting, and emotion decoding abilities [26,27,28,29,30]. These apparent cognitive deficits may contribute to the perpetration of family violence in a number of ways, but little is known about how these cognitive deficits are specifically related to the acceptability of IPVAW. Although the question of how cognitive impairments may facilitate violence expression remains unanswered, exploring these basic cognitive processes may help to explain why individuals who present cognitive dysfunctions are more prone to violence than normative individuals. 

Executive dysfunctions entail disruptions in planning logically, adapting behavior to demanding contexts, engaging in mental flexibility, and inhibiting inappropriate behaviors, among others [26,31,32,33]. Higher-order cognitive skills, such as executive functioning and social cognition, directly regulate human behavior (e.g., inhibition of automatic motor responses, coping strategies) [34]. However, other basic cognitive processes, such as memory and attention, which sustain adequate functioning of higher-order cognitive skills, are still an important area of study. 

Consistent with social-cognitive theories of intimate partner violence [35,36], these cognitive deficits may serve to lower the threshold for violence expression, particularly in response to ambiguous contexts and/or stimuli [37]. For example, some studies indicate that IPVAW perpetrators with low mental flexibility and poor emotion decoding abilities display more sexist stereotypes in comparison with individuals who perform better on these two neuropsychological tests [38,39]. However, thus far, no studies have investigated whether individuals with these cognitive deficits are also more likely to justify or accept IPVAW. The present study explores whether these cognitive deficits are directly related to the acceptability of IPVAW, which could indicate whether modifying such cognitive deficits might be an important intervention strategy to decrease the acceptability of IPVAW.

Drawing on the above, the current study examines the relationship between information processing deficits, such as attention switching, set-shifting, and emotion decoding abilities, and attitudes of acceptability of IPVAW, while controlling for the effects of age, educational level, and drug abuse. These cognitive abilities have demonstrated important age-related declines in later adulthood [40,41,42]. Furthermore, formal education and drug abuse may also contribute to impaired cognitive processing [43,44]. Because IPVAW perpetrators are a heterogeneous sample, it is appropriate to control for the role of these potential confounds [40,41,42,43,44]. Regarding measurement issues, in this study, we used neuropsychological tests to assess cognitive skills and an analog task to measure the acceptability of IPVAW. Neuropsychological tests have greater diagnostic value than self-reports when cognitive deficits or brain injuries might be present [45,46]. Furthermore, measuring the acceptability of IPVAW is also challenging, particularly in offenders, because response biases, such as social desirability or response distortions arising from a fear of negative consequences, complicate its measurement [14,23,47,48]. Self-reports are especially vulnerable to response distortions. On the other hand, implicit measures minimize this type of bias when assessing sensitive issues, such as the acceptability of violence in intimate relationships [14,23,48,49,50]. To this end, the present study used an analog task developed specifically to provide a more implicit measure of the acceptability of IPVAW [14]. We hypothesized that greater cognitive deficits would be related to greater acceptability of IPVAW in a sample of male perpetrators, after controlling for potential confounds.

## 2. Methods 

### 2.1. Participants

IPVAW perpetrators were recruited from the CONTEXTO psycho-educational and community-based treatment program (a mandatory program for adjudicated male abusers) administered by the Department of Social Psychology at the University of Valencia from April 2015 to May 2017. Initially, of a total sample of 131 IPVAW perpetrators, 94 participants agreed to participate in the study. Of the eligible IPVAW perpetrators identified, 84 volunteered for this study and completed the assessment. In fact, ten participants were removed from the study because they did not complete the assessment (e.g., refused to participate during the study) and/or they dropped out of the intervention before starting. The IPVAW perpetrators had been sentenced to less than two years in prison but had no previous criminal record. Therefore, their sentence had been suspended on the condition that they attend this type of intervention program [51,52,53]. Sample characteristics (socio-demographics and drug use characteristics) are described in Table 1.

All the participants were right-handed, healthy, and living in Valencia, Spain. Participants were properly informed about the research protocol and provided their written informed consent. Furthermore, the experiment was performed in accordance with the Helsinki Declaration and approved by the University of Valencia Ethics Committee (H1348835571691).

### 2.2. Procedure

Participants were initially informed that if they refused to participate in our study, this refusal would not affect their legal situation. Moreover, they were assured that the judicial system would not have access to individual responses provided during the research.

Each participant engaged in three sessions in the psychology laboratories of the University of Valencia before starting the intervention program. In fact, this was part of the initial assessment for the CONTEXTO intervention program. In the first session, participants were interviewed in order to identify individuals who had any severe organic or psychological disorders or diseases (participants were excluded if any of these conditions were present). Two clinical psychologists trained in IPVAW treatment initially interviewed the participants to detect psychopathology and personality disorders. The content of this interview was designed by our research team, based on the psychopathological dimensions evaluated by the DSM-5 criteria. These two independent psychologists conducted two different qualitative interviews with each participant, and inter-rater agreement was assessed by Cohen’s kappa. Furthermore, the qualitative interview was complemented by self-reports, such as the Symptom Checklist-90-Revised (SCL-90-R) and the Millon Clinical Multiaxial Inventory-III (MCMI-III). Participants were removed from the study if the qualitative interviews and self-report scores detected mental and/or personality disorders. Additionally, participants’ drug use (i.e., alcohol, marijuana, and cocaine) was assessed in terms of the frequency of consumption and the amount consumed, using AUDIT to check for the frequency of alcohol use and the substance dependence severity scale for marijuana and cocaine. We focused on these drugs because they are the ones most consumed by this population. In the second session, after arriving at the laboratory, data were collected on demographic and anthropometric characteristics. In addition, participants completed the PVAM (see below) on a computer. The third session took place one day after the second session, between 10 a.m. and 2 p.m., to avoid fatigue effects that might compromise cognitive performance after a workday. In this session, three neuropsychological tests were administrated: the attention switching task (AST) (a computerized test), the Wisconsin card sorting test (WCST), and the “Reading the Mind in the Eyes” (eyes test). 

### 2.3. Measures

#### 2.3.1. Partner Violence Acceptability Movie Task (PVAM)

This task was created following Rodriguez and colleagues’ (2011) procedure (see [11] for details). This task consists of a series of clips (90 seconds each) obtained from Spanish commercially dubbed films that depict male physical aggression toward the female partner during the episode. Participants were asked to stop the scenes when they thought the man had become too violent. Participants’ reaction time to video scenes of violence (measured in fractions of seconds) were employed to assess acceptability of intimate partner violence against women, with slow reaction times interpreted as higher tolerance or acceptability of IPVAW. The PVAM provides an alternative to questionnaires, which are usually affected by participant distortions, in order to study to what extent IPVAW perpetrators maintain biases and tolerate certain forms of violence. PVAM delay scores demonstrate adequate internal consistency and correlate with a self-report measure of attitudes and beliefs about IPVAW, thus demonstrating construct validity [11]. 

#### 2.3.2. Neuropsychological Measurements

The Attention Switching Task (AST) measures an individual’s ability to switch his/her attention between the direction of an arrow and its location on the screen while avoiding distracting events. It is a highly cognitively demanding test because participants need to switch their attention between congruent (e.g., arrow on the right side of the screen pointing to the right) and incongruent (e.g., arrow on the right side of the screen pointing to the left) stimuli. The score of interest considered in this study was the percentage of correct responses [54].

The revised Spanish version of the Wisconsin card sorting test (WCST) [55] was used to measure cognitive flexibility. Cards have to be sorted until six categories are matched or until all 128 cards are sorted. Cards are matched according to different criteria, such as color, form, and number. After 10 consecutive correct cards are sorted, a new criterion is instituted without warning. The dependent variables included in this study were: number of correct responses, number of errors, number of perseverative errors, and number of categories completed.

The revised version of the Reading the Mind in the Eyes [Eyes Test, 56] was administered. This task is considered an advanced theory of mind test that measures the emotion decoding process. The eyes test contains 36 black and white photographs of the eye region of the faces of different actors and actresses. Participants must attribute the mental state of the actors by choosing which of four words best describe what the person in the photo is thinking or feeling. Scores are calculated as the total number of correct selections for all 36 photographs [56]. 

### 2.4. Drug Abuse

The Spanish version of the Alcohol Use Disorders Identification Test (AUDIT) [57] was used to identify participants’ alcohol consumption habits. The scale consists of 10 self-report items ranging from zero (never) to four (daily or almost daily). Item scores contribute to a total score ranging from 0 to 40. Total scores of eight or above are recommended indicators of hazardous and harmful alcohol use, as well as possible alcohol dependence. Cronbach’s alpha in this study was 0.90.

The Severity of Dependence Scale (SDS), adapted to Spanish [58], was used to assess participants’ substance abuse (e.g., cocaine, marijuana). On this short, five-item scale, items are rated from zero (never/almost never) to three (always/nearly always). A total SDS score can be obtained by adding up the scores on all the items, with higher total scores indicating higher levels of dependence. According to these total scores, participants were classified as zero (non-abuser) or one (abuser). Moreover, self-reported drug consumption per week was recorded. 

Impulsivity was measured with the Spanish version [59] of the Plutchik Impulsivity Scale [60]. This self-report assesses impulsivity as an immediate reaction without considering any consequences of the behavior. This test consists of 15 items with scores ranging from one (= never) to four (= almost always). The Cronbach’s alpha in this study was 0.82.

### 2.5. Data Analysis

Six linear regression models were conducted to investigate whether the cognitive deficits (attention-switching, cognitive flexibility, and emotional decoding abilities) predicted attitudes of acceptability of IPVAW (PVAM scores), including socio-demographic variables (age, educational level), drug abuse, and self-reported impulsivity as covariates. All statistical analyses were performed with SPSS 17.0 for Windows (SPSS Inc., Chicago, IL, USA), with the alpha level set at 0.05.

## 3. Results

IPVAW perpetrators’ mean age was 39.88 years old (SD = 11.35). The majority were Spanish, divorced or single, and presented a primary educational level.

**Attention-switching (AST percent), set-shifting (WCST), and emotion decoding abilities as predictors of attitudes of acceptability of IPVAW.** Deficits in attention-switching, set-shifting, and emotion decoding abilities significantly predicted higher acceptability of IPVAW. Specifically, the percentage of correct answers on the AST predicted 0.197 of the variance in attitudes of IPVAW acceptability scores (*β* = −0.455, *p* < 0.001; *F*= 20.13, *p* = 0.001, 95% CI = −0.29 to −0.11). After including the participants’ age, educational level, drug abuse (alcohol and marijuana), and self-reported impulsivity, the prediction of PVAM scores remained significant (*β* = −0.443, *p* < 0.001, 95% CI = −0.29 to −0.10). Additionally, on the WCST, the number of errors and the number of categories completed predicted 0.046 and 0.006, respectively, of the variance in attitudes of acceptability of IPVAW (*β* = −0.269, *p* = 0.021; *F* = 5.56, *p* = 0.021, 95% CI = −1.32 to −0.11 and *β* = −0.269, *p* = 0.021; *F* = 5.56, *p* = 0.021, 95% CI = −1.32 to −0.11, respectively), and they remained significant after including covariates (*β* = −0.263, *p* < 0.05, 95% CI = −1.33 to −0.09 and *β* = −0.263, *p* < 0.05, 95% CI = −1.33 to −0.09, respectively). Finally, the eyes test scores predicted .0095 of the attitudes of acceptability of IPVAW (*β* = −0.325, *p* < 0.01; *F*= 9.68, *p* = 0.003, 95% CI = −0.79 to −0.18). As in the previous cases, after controlling for covariates, the prediction remained significant (*β* = −0.271, *p* < 0.05, 95% CI = −0.29 to −0.10). 

## 4. Discussion

The current study evaluated whether cognitive deficits are associated with more favorable IPVAW attitudes. As hypothesized, the data indicate that, among IPVAW perpetrators, attention switching, set-shifting, and emotion decoding abilities were significantly related to attitudes of acceptability of IPVAW, with poorer cognitive performance negatively associated with IPVAW acceptability (longer delays in stopping the videos). Moreover, these results remained significant after controlling for the effects of several potential confounds, including socio-demographic variables, such as age and educational level, as well as drug consumption. 

Our preliminary results suggest that poor performance on neuropsychological tests, which might signal impairments in basic cognitive processes, may predict higher acceptability of IPVAW (longer delay times until stopping the PVAM task). Poor cognitive functioning has been postulated as a relevant risk factor for violence recidivism in forensic populations [61,62,63]. 

Although little is known about how cognitive deficits facilitate violence perpetration, several models strive to explain this relationship. A deficit in the ability to focus and concentrate one’s attention involves less openness to key environmental signals, which is consistent with classic socio-cognitive theories [64,65] where executive dysfunction has been linked to poor emotion regulation [34]. Moreover, as emotion decoding skills partially sustain emotional and behavioral regulation [66], empathy deficits could lead to the misinterpretation of others’ intentions, facilitating the onset of violence if the individual does not have an adequate regulatory system [27,67]. Indeed, poor empathy, including emotion decoding deficits, was found to increase the risk of physical child abuse by Spanish IPVAW offenders [68], and violent offenders performed worse on facial-affect recognition than non-violent offenders [69]. With regard to partner violence, individuals with these cognitive deficits might spend more time attempting to detect specific environmental signals in the video scenes of the analog task (e.g., actors’ facial expressions, body language)—deficits that could precede violence perpetration during couple conflict. Indeed, an integrative, biopsychosocial approach, such as social information processing theory, provides a framework for the way social cues are decoded in IPV situations [36]. Dysfunctional social information processing, including a diminished ability to adopt the perspective of others, could partially explain the reduced ability to rapidly detect IPVAW signals in video scenes.

This study may help to understand the processes involved in IPVAW acceptability and justification, namely, cognitive aspects that might underlie the acceptability of IPVAW. These results are also based on reaction times and neuropsychological measurements that assess the potential underlying processes influencing attitudes of acceptability of IPVAW. Taken together, these findings highlight the importance of assessing these variables before starting IPVAW intervention programs, in order to improve their effectiveness. However, these results should be included in a broader model that also considers contextual factors in order to better understand a phenomenon as complex as IPVAW. Nevertheless, this study has some limitations. An important limitation is the cross-sectional, non-experimental design, based on regression analysis, which cannot address causality. Furthermore, the limited sample size decreased statistical power. Moreover, future studies should assess the role of other drugs and/or addictions, in addition to alcohol, marijuana, and cocaine, which might play an important role in the relationships assessed in this study. Additionally, only a selection of neuropsychological tests that might contribute to IPVAW acceptability was examined, whereas many other cognitive deficits may also contribute to these attitudes. In addition, internal validity was limited by the absence of a control group to compare the measured variables and relations. In fact, it would be interesting to study whether the absence of these neuropsychological deficits translates into better PVAM scores.

Our data suggest several important avenues for future research, including the identification of other neuropsychological deficits that might moderate or mediate the relations between IPVAW acceptability and the risk of recidivism. Furthermore, the results of this research could benefit rehabilitation programs designed for IPVAW offenders, particularly those focused primarily on changing beliefs, biases, and cognitive distortions of offenders. Our findings provide evidence supporting the creation of new directions for intervention programs, focused on strengthening basic emotional decoding processes and addressing cognitive deficits. These adjunct programs, based on extensive cognitive practice and strategies, may help IPVAW perpetrators to acquire new skills and compensatory mechanisms for resolving troubling and/or novel situations. Indeed, prior work on aggressive behavior observed that attention and memory skills are critical for executive functioning [26,31,32,33]. Therefore, mitigating these deficits directly can impact higher-order cognitive processes and behavior regulation. Moreover, it would be interesting to study whether improvements in basic information processing can directly diminish sexist stereotypes and/or attitudes about IPVAW acceptability. Efforts to modify these attitudes continue to be a critical aim of IPVAW offender intervention programs, given the persistent societal burden of intimate partner violence.

## Figures and Tables

**Table 1:** Mean ± SD and percentages of sociodemographic and drug use variables of participants.

Age (Years)		IPV Perpetrators (*n* = 84)
		39.88 ± 11.35
Nationality	Spanish	77.9%
	Latin Americans	9.7%
	Africans	6.4%
	Eastern European Countries	6%
Marital status	Married/Cohabiting	15.5%
	Divorced/Single	84.5%
Educational level	Primary/lower secondary	57.7%
	Upper secondary/vocational training	36.1%
	College	6.2%
Employment status	Employed	52.6%
	Unemployed	47.4%
Self-reported impulsivity		32.81 ± 6.34
AUDIT_total score (alcohol)_		4.31 ± 5.73
Cannabis	Yes	31.6%
	No	68.4%
Number of joints of cannabis per week		5.10 ± 8.37
Cocaine	Yes	0%
	No	100%
